# The right to practice medicine without repercussions: ethical issues in times of political strife

**DOI:** 10.1186/1747-5341-7-11

**Published:** 2012-09-13

**Authors:** Leith Hathout

**Affiliations:** 1 Department of Biology, Stanford University, P.O. Box 11042, Stanford, CA, 9430-–1042, USA

**Keywords:** Medical neutrality, Bahrain, Syria

## Abstract

This commentary examines the incursion on the neutrality of medical personnel now taking place as part of the human rights crises in Bahrain and Syria, and the ethical dilemmas which these incursions place not only in front of physicians practicing in those nations, but in front of the international community as a whole.

In Bahrain, physicians have recently received harsh prison terms, apparently for treating demonstrators who clashed with government forces. In Syria, physicians are under the same political pressure to avoid treating political demonstrators or to act as informants against their own patients, turning them in to government authorities. This pressure has been severe, to the point that some physicians have become complicit in the abuse of patients who were also political demonstrators.

This paper posits that physicians in certain countries in the Middle East during the “Arab Spring,” specifically Syria and Bahrain, are being used as both political pawns and political weapons in clear violation of Geneva Convention and World Medical Association guidelines, and that this puts them into the most extreme sort of “dual loyalty” dilemma. They are being forced to choose between their own safety and well-being and that of their patients – a negative sum scenario wherein there is no optimal choice. As such, an international call for a United Nations inquiry must be made in order to protect the neutrality of medical care and personnel during times of armed conflict.

## Introduction

In Western nations, it is clear that there is a significant interplay between politics and the practice of medicine. The political process, in some sense, affects both the regulations upon, and the remunerations for, the provision of healthcare. For example, in the United States, during the Supreme Court session which opened this October, the Court is expected to decide on whether physicians and patients can sue states for limiting Medicaid payments, and to hear a constitutional challenge to President Obama’s healthcare reform.

However, over the past several months, political dramas of a very different sort have impacted physicians in some countries of the Middle East. The so called “Arab Spring,” wherein ordinary citizens have risen up against their autocratic governments – such as in Tunisia, Egypt, Libya, and Syria – has underscored some of the ethical dilemmas and human rights issues which face physicians working in regions of political turmoil.

## Discussion

This commentary will highlight recent events in Bahrain and Syria, using these two nations as case studies in what sometimes happens when the long arm of political repression reaches into the hospital ward. The ethical dilemmas facing physicians in these countries essentially “speak for themselves,” and are presented to raise awareness and engender reflection. As background, it is important to note that clear and unequivocal international standards have long been established regarding the treatment of physicians during times of political strife. These rules, reflecting the norms of the Geneva Convention, are summarized in the World Medical Association (WMA) guidelines. Article 5 of the “WMA Regulations in Times of Armed Conflict,” states:

“Governments, armed forces and others in positions of power should comply with the Geneva Conventions to ensure that physicians and other health care professionals can provide care to everyone in need in situations of armed conflict. This obligation includes a requirement to protect health care personnel.”

### Article 8 of the same guidelines more pointedly states

*“Physicians have a clear duty to care for the sick and injured. Provision of such care should not be impeded or regarded as any kind of offence. Physicians must never be prosecuted or punished for complying with any of their ethical obligations*[1]*.”*

Specific suggestions for some action items in the face of these dilemmas are presented at the end of the commentary.

It must be noted that these incursions against medical personnel have been quite unique to Bahrain and Syria, and have not occurred in the remaining Arab nations during the protests of the Arab Spring. Perhaps the unifying factor in both of these cases is a very centralized authority in the head of state.

By way of brief historical review, Bahrain is a small monarchy which gained its independence from the United Kingdom in 1971. It is ruled by King Hamad bin Isa Al-Khalifa, and the king has significant authority, such as commanding the army, appojnting the Prime Minister and his cabinet, appointing the upper house of parliament and dissolving its lower house. The Prime Minister, Khalifa ibn Salman Al-Khalifa is the king’s uncle.

In Syria, President Bashar al-Assad took the reins of power in 2000 after the death of his father, Hafez al-Assad, who had ruled Syria for 30 years. After the death of Hafez al-Assad, the Syrian Parliament quickly amended the constitution to reduce the minimum presidential age from 40 to 34, making Bashar eligible for the presidency, where he was elected, as an unopposed candidate, by over 97% of the vote, according to Syrian authorities. In Syria, Bashar al-Assad also has extraordinarily strong ties to the military, such that soldiers who refused to shoot civilian demonstrators in the 2011–2012 uprisings were summarily executed by the military.

Therefore, in both nations, the ruler has significant control of the armed forces, which have been instrumental in quelling the civilian uprisings. Thus, while rulers have fallen in Tunisia, Libya, Egypt and Yemen, and some reforms have happened in Morocco, Jordan and Oman, the leaders of Bahrain and Syria have thus far been successful in retaining their hold on power. This may be because of both their willingness and ability to use more brutal measures against their populations, such as violating medical neutrality.

### I. Bahrain

The “Arab Spring,” has also come to Bahrain. There, the population has begun to revolt against the ruling monarchy, and in the ensuing political upheaval, physicians and nurses have become a significant target of political repression and frank human rights violations. Several months ago, several dozen doctors and nurses were arrested, including some Irish-trained physicians practicing in Bahrain. Their crimes? The medical personnel are officially charged with incitement to overthrow the regime. They contend, however, that their only crime was treating political demonstrators after they were injured in clashes with the monarchy’s police forces [2].

After months in prison, the doctors and nurses were released in early September. This came, at least partially, as the result of an international outcry over their arrests, and after Irish and UK physicians started a rolling fast to support a hunger strike undertaken by the imprisoned medics [3]. Although the charges were not dropped, and a court was to try their case in late September, their release was seen as a sign of hope that the government of Bahrain may be mitigating its stance, and that they would be found innocent. Such hopes proved unfounded, as on September 29^th^, the Bahrain courts convicted the 20 physicians and nurses and levied harsh sentences against them, with thirteen doctors receiving fifteen year prison terms, and the remainder getting sentences between five and ten years [4]. The physicians were convicted of a variety of offenses, including allegations of stockpiling weapons in the Salmaniya hospital and of “fabricating stories to disturb public security.”

Certainly, physicians are no strangers to human rights crises. They often put themselves in harm’s way as a part of indigenous or international relief efforts. Alternatively, individual physicians may suffer political repression as any ordinary citizen does under a repressive regime. However, what has transpired in Bahrain is somewhat unique in the modern era. The doctors claim that medical personnel are being punished solely for practicing the healing arts, ardently maintaining that their activities were entirely apolitical in nature. Dr. Ali Al-Akri, one of the imprisoned physicians, told The Guardian, “We were as far away from politics as you could be but we found ourselves in the centre of it because we were treating the victims [5].” In another statement released by the medics after the court ruling, they said, “During the times of unrest in Bahrain, we honored our medical oath to treat the wounded and save lives. And as a result, we are being rewarded with unjust and harsh sentences [6].”

The policy of the Bahrain government has drawn significant international criticism. In Geneva, the spokesman for the U.N. Human Rights Office, Rupert Colville, said that there are “severe concerns” about the sentences against the doctors and nurses. Meanwhile, Hans Hogrefe, chief policy officer for the advocacy group Physicians for Human Rights, in a statement on the group’s website said, “To imprison them as part of a political struggle is unconscionable.” Also, the president of the World Medical Association, Wonchat Subhachaturas, said that these sentences were “totally unacceptable.” He continued: “It is a sad day for medicine when physicians are incarcerated for treating patients… The disproportionate nature of the sentences handed down in this case … is a disgrace and must be overturned [7].”

It is important for Western physicians and academicians, especially those involved in the study of ethics and philosophy, to add their voice to this chorus of international concern. This is so for a variety of reasons. Firstly, the stature of the Western nations in general, both as political and economic powers and as scientific leaders in the arena of medicine, would lend significant gravitas to the cause of Bahrain’s beleaguered physicians. This support is critical, particularly given the seeming difficulty of getting medical societies to offer formal statements regarding the situation. For example, Professor Damian McCormack, an Irish Orthopedic Surgeon at Temple Street Children’s University Hospital, shared with the Irish Medical Times some of his frustrations when he attempted to galvanize support for the some of the Bahraini physicians he had trained, and who were now behind bars: “[I] wrote to several organisations … who were not helpful [8].”

Secondly, the coming few weeks represent a critical time in deciding the ultimate fate of the convicted physicians and nurses in Bahrain. They have called upon U.N. Secretary-General Ban Ki-moon to review their case and are attempting to appeal their verdicts in Bahrain’s court system. Without strong international support, many have lost hope that the appeals process will yield any results. In an especially heart-rending story in the Associated Press, Dr. Nada Daiif, one of the convicted physicians, spoke of her heartbreak as she stroked the hair and touched the cheeks of her two young children, trying to spend as much time with them as possible before having to report for her fifteen year sentence, and of her lack of faith in the appeals process, … “…we know very well they are using our case as a political card against the opposition [6].”

### II. Syria

An even more egregious example of the assault on medicine which can take place in times of political strife is now playing out in Syria. In a report released October 25^th^ , titled “Health Crisis: Syrian Government Targets the Wounded and Health Care Workers,” Amnesty International alleges a wide range of human rights violations against both patients and doctors, in a continuing attempt to quell the on-going uprising against President Bashar al-Assad’s government [9]. Amnesty states that “The Syrian government has turned hospitals into instruments of repression in its efforts to crush opposition [10].”

The problems in Syria are manifest at three levels. The first is that the sanctity of hospitals as politically neutral arenas has been violated. Amnesty reports that “It is deeply alarming that the Syrian authorities seem to have given the security forces a free rein in hospitals [10].” The report details that security forces now enter the government-run hospital wards looking for wounded protestors, who may then be removed from the hospital, without regard to their injuries. Cilina Nasser, an Amnesty International researcher on the Middle East and North Africa has told news sources that doctors reported to her that sometimes they would be treating a wounded protestor, and that “the following day they would come to the hospital … to check on this wounded patient and they find out that the security forces have already removed them from there and they took them to an unknown destination [11].” One particularly disturbing example is a story related by Amnesty, that on September 7th, security forces looking for a protestor opposed to the government raided al-Birr wa al-Khadamat Hospital in Homs. When they did not find him, they arrested 18 wounded people.

This situation has apparently forced many of the wounded to avoid care in state hospitals, for fear of abuse, arrest or torture. A BBC report, highlighting the work of Amnesty International, quotes a medic as saying, “Given the scale and seriousness of the injuries being sustained by people across the country, it is disturbing to find that many consider it safer to risk not having major wounds treated rather than going to proper medical facilities [12].” Thus, doctors have noted with alarm that despite the spiraling death rates among political protestors, (the United Nations estimates about 3000 so far), there has been a significant decrease in hospital admissions for trauma or gunshot wound injuries since May.

For those patients who choose to come to the hospital, doctors are forced to try to work surreptitiously on their behalf, as illustrated by the so-called “blood dilemma.” In Syria, blood supplies are administered centrally, and this administration is under the control of the Ministry of Defense. Thus, the doctor’s predicament is well summarized by one physician, who states, “We faced a dilemma every time we received a patient with a firearm injury and an urgent need of blood: if we send a request to the Central Blood Bank, the security would know about him and we would be putting him at risk or arrest and torture, and possibly death in custody [10].”

A second problem is the intense pressure facing Syrian doctors in their treatment of patients, with doctors themselves having to risk reprisals for treating wounded protestors. Amnesty reports that “Hospital workers suspected of treating protesters and others injured in unrest-related incidents have themselves faced arrest and torture [10].” For example, on August 7^th^, soldiers raided a hospital in Homs, arresting 7 hospital workers. One doctor spoke to Amnesty International about the interrogation process, where some of his colleagues were badly beaten:“[The interrogator] asked: ’do you want to be tortured or do you want to talk?’ … He accused me and my colleagues of treating the wounded without reporting them to the authorities, and asked me for the names of the wounded [10].”

Third, and most disturbing of all, is the charge that the Syrian government has made, either by direct or implied threat, some of the doctors and nurses working in state-run hospitals complicit in the abuse and torture of patients. In a BBC report, Ms. Nasser is reported as saying, ‘In many cases hospital staff appear to have taken part in torture and ill treatment of the very people they are supposed to care for [12].’ The Amnesty International investigation found that patients have been abused by both medical staff and security personnel in at least four hospitals: the National Hospitals in Banias, Homs and Tell Kalakh and the military hospital in Homs. According to Amnesty, one doctor at Homs military hospital told Amnesty International he had seen four doctors and more than 20 nurses abusing patients [10]. For example, an earlier report by Amnesty International (July 6, 2011) documents the treatment of Wassim, a 21-year-old protester in the Syrian town of Talkalakh. After an injury from a soldier’s bayonet, Wassim was taken to al-Bassel hospital, which had been occupied by Syrian security forces. He was abused by the hospital staff, tied up and had alcohol splashed in his face, until he was unable to breathe. He reported that then, ‘The nurses, men and women […] swore at me and beat me hard and one female nurse punched me repeatedly with all her strength on my chest. Some were taking off their shoes and slapping me with them. I could hear many voices asking: ’You want freedom, eh? [13]” The report states he later had his wounds stitched without anesthesia. Another story is relayed to the BBC by a doctor named Ahmad, who tells of alerting the hospital manager after seeing a 14-year-old, who was brought in with bullet wounds, being beaten up by a nurse. After lodging his complaint, he received a request to report to the security authorities, after the nurse told officials that he was part of “an Islamic organization.” He declined to report, and chose instead to leave Syria [12].

However, not all doctors can or would make such a choice. Under the intense pressure, some have seemingly decided that it may be safer to be on the side of the state than the patient. Thus far, these doctors have faced no reprisals. In an opinion piece in Al-Jazeera English, Rajaie Batniji , a Stanford physician and an affiliate of the Center on Democracy, Development and Rule of Law at Stanford University, calls for banning such physicians from the profession, asking: “… in their pursuit of perceived enemies of the state, have these physicians become enemies of the profession? Doctors involved in torture should be pursued as enemies of medicine: their crimes documented, their professional credentials revoked, and their ability to practice internationally thwarted [14].”

While it may seem that doctors facing arrest or torture have little choice but to be complicit in the crimes of their repressive government, it must be made clear that the majority of Syrian doctors have resisted the pressure, and in the words of Dr. Batniji, have “acted heroically.” For example, in a CNN story titled “Syria’s ’secret doctors’ risk their own lives,” we learn of a group called “Damascus doctors,” physicians who run secret clinics for patients too afraid to go to the state hospitals [15].

## Conclusion

Clearly, there are many physicians practicing in Bahrain and Syria who have presumably treated the sick and injured without arrest. Likewise, most physicians in Syria have honored their oaths, and have avoided involvement in the abuse of patients, or even in identifying them to authorities as “enemies of the state.” However, the manner in which these cases have unfolded warrants deep concern and serious review, preferably with international oversight. On the face of it, there appears to be several serious violations, if not flouting, of international standards regarding the treatment of physicians during times armed conflict. These violations have had a genuine human cost in terms of the welfare of both physicians and patients. Moreover, they have put medical personnel before the most extreme sort of dual loyalty dilemma, such that even if they do not suffer physical harm, they are likely to suffer the psychological trauma that comes from an inescapable ethical predicament. Several interesting papers have been written recently on the issue of double loyalty in medicine during armed conflict analyzing the conduct of physicians in the Abu Ghraib and Guantanamo Bay prisons, where-in physicians were pressured to be complicit in the torture of prisoners. For example, Clark argues that in these cases, physicians were placed in a dual loyalty conflict between the needs of their patients, who were political detainees, and their employer, the military. Clark concludes that the “United States military medical system failed to protect detainee’s human rights, violated the basic principles of medical ethics and ignored the basic tenets of medical professionalism [16].” A similar conclusion was reached by Professor Steven Miles in a 2004 Lancet article [17].

However, in those cases, the dilemma is less severe for physicians than in the cases we have been discussing in the Middle East. The physicians of the US military could rationalize that they are violating the rights of hostile enemy combatants, and that the benefit of information gained under harsh interrogation may outweigh the harm of violating the rights of their patients. This argument was made in a “point-counterpoint” discussion in the Cambridge Quarterly of Healthcare Ethics. In that article, a point of view was posited that the aim of the military physician is to identify with the goals of the military to best protect their citizenry and that “This best protection unequivocally requires armed forces having military physicians committed to doing what is required to secure victory [18].”

In the cases of Syria and Bahrain, however, physicians cannot have the solace of this point of view. They are being asked to aggress upon their fellow citizens. If they refuse, they face prison or torture, rather than the more implicit pressures facing American military physicians in Abu Ghraib or Guantanamo Bay. Various authors who have analyzed the risks physicians face in times of political turmoil or military struggle have suggested that physicians should honor their ethical obligations even if this jeopardizes them personally [19,20].

However, as noted by Benatar and Upshur, “Upholding a heroic standard in all circumstances is unreasonable and therefore unrealistic [21].”

The real answer to this ethical dilemma is not to ask doctors to risk their life and liberty to honor their professional vows. Rather, to safeguard the welfare of both patients and doctors, and to honor the efforts of the many doctors putting themselves at risk on behalf of their patients, it is important to follow the recommendations made in the recent excellent Lancet article by Len Rubenstein and Melanie Bittle, titled “Responsibility for protection of medical workers and facilities in armed conflict [22].” In that article, they call for, among other things, an expanded mandate for WHO, including a UN Security Council resolution, allowing it to investigate and document abuses against the medical profession, whether they are committed by security forces or by physicians. Also, it seems important that the American and European medical establishments support the requests of the Bahrain physicians for an independent review of their case, such as by the UN. Additionally, the United Nations’ Human Rights Council should utilize its Special Procedures mandate to appoint a UN Special Rapporteur to investigate and report on violations of the World Medical Association Guidelines and the Geneva Convention in Bahrain, Syria and other similarly affected countries. Given the prominent role of former UN secretary-general Kofi Annan in current efforts to stop the bloodshed in Syria, it is an opportune time for the international body to spotlight the issue of physicians being used as pawns of war. At the time of this writing, the United Nations is discussing the deployment of UN observers as monitors of the fledgling cease-fire agreement between the Syrian government and the opposition. As such, this would be precisely the opportune moment for a UN Special Rapporteur to be deployed along with the cadre of United Nations’ monitors. Such international efforts will allow a more in depth review to assess whether indeed the status of practicing physicians as neutral non-combatants in times of political strife has been violated. If such is the case, it would have serious repercussions for physicians on a large scale, and for a long time to come.

## Abbreviations

WMA: World Medical Association; WHO: World Health Organization.

## Competing interests

The author(s) declare that they have no competing interests.
